# Correction: Determinants of HIV testing among Filipino women: Results from the 2013 Philippine National Demographic and Health Survey

**DOI:** 10.1371/journal.pone.0246013

**Published:** 2021-01-26

**Authors:** Veincent Christian F. Pepito, Sam Newton

There are errors in the Abstract Results. The following values published in the Abstract Results are incorrect: richer (aOR = 3.00; 95% CI: 1.37–5.68), and richest (aOR = 4.14; 95% CI: 1.80–5.91) populations. The corrected values are as follows: richer (aOR = 3.00; 95% CI: 1.37–6.58), and richest (aOR = 4.14; 95% CI: 1.80–9.51) populations.

There are also errors in the final paragraph of the Results section on page 9. The following values published in this paragraph are incorrect: richer (aOR = 3.00; 95% CI: 1.37–5.68), and richest (aOR = 4.14; 95% CI: 1.80–5.91) populations. The corrected values are as follows: richer (aOR = 3.00; 95% CI: 1.37–6.58), and richest (aOR = 4.14; 95% CI: 1.80–9.51) populations.

In the Data management subsection of the Methods section, there are errors in the second sentence of the third paragraph. The listed questions erroneously link to references. The questions should instead be linked as follows: HIV knowledge score were derived from the following questions: [a] Ever heard of AIDS; [b] Reduce risk of getting HIV: Always use condoms during sex; [c] Reduce risk of getting HIV: have one sex partner only, who has no other partners; [d] Can get HIV from mosquito bites; [e] Can get HIV by sharing food with person who has AIDS; [f] A healthy looking person can have HIV; and [g] Can get AIDS by shaking hands. Tolerance to domestic violence score was aggregated from the following questions: [a] Beating justified if wife goes out without telling husband; [b] Beating justified if wife neglects the children; [c] Beating justified if wife argues with husband; [d] Beating justified if wife refuses to have sex with husband; [e] Beating justified if wife burns the food. Women’s empowerment score was derived from the following questions: [a] Who decides on your healthcare; [b] Who decides on large household purchases; [c] Who decides on daily household purchases; [d] Who decides on visits to family or relatives; and [e] Who decides what to do with money husband earns.

There are also errors in the confidence intervals of Number of lifetime sexual partners and Women’s empowerment score in Table 2. Please find the corrected Table 2 below.

**Table 2:** Description of study participants and crude associations between quantitative exposure variables and HIV testing.

Variable	Number (%) of observations with data	Range	Mean and 95% Confidence Interval (CI)	Median	Distribution	Rank-sum test p-value	OR^a^	p-value
Number of children	16,155 (100)	0–19	2.06 (2.01–2.11)	1	Right-skewed	0.07	1.00 (0.96–1.04)	0.91
Number of lifetime sexual partners	16,145 (99.9)	0–95	0.76 (0.74–0.79)	1	Right-skewed	<0.01	1.14 (0.95–1.37)	0.15
HIV knowledge score	14,607 (90.4)	1–7	4.53 (4.51–4.57)	5	Left-skewed	0.02	1.08 (0.98–1.18)	0.10
Tolerance to domestic violence score	16,144 (99.9)	0–5	0.26 (0.24–0.28)	0	Right-skewed	0.52	0.98 (0.88–1.11)	0.80
Women’s empowerment score	9,456 (58.5)	0–10	6.50 (6.45–6.54)	6	Left-skewed	0.68	1.03 (0.95–1.12)	0.52

^a^ Denote increase in odds of HIV testing per unit increase in the value of the quantitative exposure
