# Supplementary material for: Determinants of HIV testing among Filipino women: Results from the 2013 Philippine National Demographic and Health Survey
Source: PLoS One. 2020 May 12;15(5):e0232620. doi: 10.1371/journal.pone.0232620 (PMC7217462; doi:10.1371/journal.pone.0232620)
Supplement: S1 Appendix — (DOCX) [file pone.0232620.s001.docx]

Supplementary Appendix 1 – Definition of Variables and Coding Manual

| Variable | Type of Variable | Variable name | Operational definition | Coding and label |
| --- | --- | --- | --- | --- |
| Age | Continuous | v012 | Age of the respondent (in years) at the time of the interview | As is |
| Age | Categorical | age | Recoded from v012 of the dataset. | 17 – 15-19 y/o  22 – 20-24 y/o  27 – 25-29 y/o  32 – 30-34 y/o  37 – 35-39 y/o  42 – 40-44 y/o  47 – 45-49 y/o |
| Educational attainment | Categorical | v106 | Highest educational attainment of the respondent at the time of the interview. May be no education, primary, secondary, or higher. | 0 – no education  1 – primary  2 – secondary  3 – higher |
| Educational attainment | Categorical | educ | Recoded from v106 of the dataset | 0 – No/primary education  1 – Secondary  2 - Higher |
| Employment status | Categorical | v731 | Whether or not the respondent is employed in the 12 months preceding the interview. May be No, In the Past Year, Currently Working, or have a job but on leave last 7 days. | 0 – No  1 – In the past  2 – Currently working  3 – Have a job but on leave last 7 days |
| Employment status | Categorical | work | Recoded from v731 of the dataset. | 0 – No (0 and 1 in original dataset)  1 – Yes (2 and 3 in original dataset) |
| Marital status | Categorical | v501 | Marital status of the respondent at the time of the interview. May either be never married, married, living together, widowed, divorced, separated. | 0 – Never in union  1 – Married  2 – Living with partner  3 – Widowed  4 – Divorced  5 – Separated |
| Marital status | Categorical | cs | Recoded from v501 of the dataset | 0 – Never in union  1 – Married  2 – Living with partner  3 – Widowed/  divorced/separated |
| Condom Use | Categorical | v761 | Whether or not the respondent used a condom during her last sexual intercourse as of the date of the interview | 0 – No 1 - Yes |
| Consistent condom use | Categorical | v833a | Whether or not the respondent consistently used condom with most recent partner for the past 12 months | 0 – No  1 – Yes |
| Condom use | Categorical | conduse | Generated from v761 and v833a of the dataset | 0 – never had any sexual partner  1 – had sexual partner/s but didn’t use condom  2 – had sexual partner/s; used condom inconsistently  3 – had sexual partner/s; used condom consistently |
| Condom access | Categorical – three categories | v769 | Whether or not the respondent can get a condom | 0 – No  1 – Yes  8 – Don’t Know |
| Condom access | Categorical - dichotomous | condacc | Recoded from v769 of the dataset. | 0 – Yes  1 – No/Don’t know |
| Current use of contraception by method type | Categorical-dichotomous | v313 | Type of contraception used by the respondent by type. May be no method, folkloric method, traditional method, and modern method. | 0 – No method  1 – Folkloric method  2 – Traditional method  3 – Modern method |
| Use of traditional or folkloric method | Categorical - dichotomous | trad | Recoded from v313 of the dataset. | 0 – No (0 and 3 in original dataset)  1 – Yes (1 and 2 in original dataset) |
| Does not use tobacco | Categorical- dichotomous | v463z | Whether or not the respondent smokes cigarette or uses any form of tobacco as of the date of the interview. | 0 – Uses tobacco  1 - Nonsmoker |
| Tobacco consumption | Categorical - dichotomous | tob | Recoded from v463z of the dataset | 0 – Nonsmoker (1 in original dataset)  1 – Smoker (0 in original dataset) |
| Age of husband/  partner | Continuous | v730 | Age of husband or partner of the respondent at the time of interview | As is. |
| Age of husband/  partner | Categorical – eight categories | agepart | Recoded from v730 of the dataset | 19 – 15-24 y/o  27 – 25-29 y/o  32 – 30-34 y/o  37 – 35-39 y/o  42 – 40-44 y/o  47 – 45-49 y/o  52 – 50+ y/o |
| Educational attainment of partner | Categorical – four categories | v701 | Highest educational attainment of the respondent at the time of the interview. May be no education, primary, secondary, or higher. | 0 – No education  1 – Primary  2 – Secondary  3 – Higher |
| Educational attainment of partner | Categorical - dichotomous | educpart | Recoded from v701 of the dataset | 0 – No/primary education  1 – Secondary  2 – Higher |
| HIV knowledge | Categorical – three categories | v751, v754cp, v754dp, v754jp, v754wp, v756, s906 | HIV Knowledge questions of the survey. May be yes, no, or maybe. | 0 – No  1 – Yes  8 – Don’t know |
| HIV Knowledge | Continuous | hivk | May range from 0-7, with higher scores implying better knowledge. In computing the score, a variable indicating whether the respondent is correct for each item was created and was given one point for each correct answer. Those who reported “Don’t Know” were classified as having wrong answers, while those who have missing responses to any of the questions would not have any HIV knowledge score. | As is. |
| Wealth index | Categorical- five categories | v190 | Wealth index of the respondent. May either be lowest, second, middle, fourth, and highest quintile. | 0 – Poorest  1 – Poorer  2 – Middle  3 – Richer  4 - Richest |
| Wealth index | Categorical – five categories | ses | Generated from v190 of the dataset. | 0 – Poorest  1 – Poorer  2 – Middle  3 – Richer  4 - Richest |
| Place of residence | Categorical-dichotomous | v025 | Whether the address of the respondent at the time of the interview is a rural area or an urban area. | 1 – Urban  2 - Rural |
| Address | Categorical - dichotomous | address | Generated from v025 of the dataset. | 1 – Urban  2 – Rural |
| Domestic violence questions of the survey | Categorical – three categories | v744a, v744b, v744c, v744d, v744e | Domestic violence questions of the survey. Responses may either be yes, no, or don’t know. | 0 – No  1 – Yes  8 – Don’t know |
| Tolerance to domestic violence | Continuous | dv | Summarized results from domestic violence toleration questions. May range from 0-5, with higher scores implying more reasons found by the respondent where gender-based violence is justified. In determining the tolerance to domestic violence score of each respondent, each of the responses was given a score of 1 for each of the items that the respondent feels that domestic violence is justified. The total score from the five items were added. Don’t know was recoded as a ‘Yes’, and is given one point as the respondents are expected to know that none of the items justifies domestic violence. Respondents with missing responses to any of the questions will not have a score. | As is. |
| Women’s empowerment | Categorical – six categories | v743a,  v743b, v743c, v743d, v743f | Women’s empowerment questions of the survey. Responses may either be respondent alone, respondent and husband/partner, husband/partner alone, or someone else. | 1 – Respondent alone  2 – Respondent and husband/partner  4 – Husband/  partner alone  5 – Someone else  6 – Not applicable  7 – Husband does not earn any income (v743f only) |
| Women’s empowerment | Continuous | we | Cumulative score of each respondent for four indicators of women empowerment, wherein the respondent is responsible for making decisions for her own healthcare, major household purchases, purchases for daily household needs, and visits to her family or relatives. May range from 0-10, with higher scores implying that the respondent is more empowered to make life choices. In computing the score for each individual, responses that are “Respondent Alone” will be coded as 2, “Respondent and husband/partner” will be coded as 1, and “Husband/partner alone”, “Other” or “Someone else” is recoded as 0. No score will be computed for those who responded “Not applicable” to any of the items or “Husband does not earn any income”, or those who have missing data for any of the items. These scores were added for each respondent to determine the women’s empowerment score. | As is. |
| Number of children | Continuous | s210 | Number of children ever born to the respondent. Includes livebirths, livebirths of children who are no longer living with the respondent, as well as stillbirths as of the date of the interview. | As is |
| Number of children | Continuous | nchildren | Generated from s210 of the dataset. | As is. |
| Religion | Categorical- seven categories | v130 | Religious affiliation of the respondent at the time of the interview. May either be Roman Catholic, Protestant, Iglesia ni Cristo, Aglipay, Islam, None, or Other. | 1 – Roman Catholic  2 – Protestant  3 – Iglesia ni Cristo  4 – Aglipay  5 – Islam  6 – None  7 - Other |
| Religion | Categorical – four categories | religion | Religious affiliation of the respondent at the time of the interview. May either be Christian–Roman Catholic, Christian–Other denomination, Islam, Other beliefs (including None). | 1 – Roman Catholic  2 – Christian Other denomination (2, 3, and 4 in the original dataset)  3 – Islam (5 in the original dataset)  4 – None/other beliefs (7 and 96 in the original dataset) |
| Reads newspaper at least once a week | Categorical-three categories | v157 | Whether or not the respondent reads any newspaper at least once a week. | 0 – Not at all  1 – Less than one a week  2 – At least once a week |
| Newspaper | Categorical -dichotomous | news | Recoded from v157 of the dataset. | 0 – Not/Less than once a week (0 and 1 in original dataset)  1 – At least once a week (2 in original dataset) |
| Watches television at least once a week | Categorical – three categories | v159 | Whether or not the respondent watches television at least once a week | 0 – Not at all  1 – Less than one a week  2 – At least once a week |
| Television | Categorical-dichotomous | tv | Recoded from v159 of the dataset. | 0 – Not/Less than once a week (0 and 1 in original dataset)  1 – At least once a week (2 in original dataset) |
| Listens to the radio at least once a week | Categorical-three categories | v158 | Whether or not the respondent listens to the radio at least once a week | 0 – Not at all  1 – Less than one a week  2 – At least once a week |
| Radio | Categorical-dichotomous | radio | Recoded form v158 of the dataset. | 0 – Not/Less than once a week (0 and 1 in original dataset)  1 – At least once a week (2 in original dataset) |
| Checks e-mail or surfs the internet at least once a week | Categorical – three categories | s111 | Whether or not the respondent surfs the internet or checks e-mail at least once a week | 0 – Not at all  1 – Less than one a week  2 – At least once a week |
| Internet | Categorical-dichotomous | internet | Recoded from s111 of the dataset. | 0 – Not/Less than once a week (0 and 1 in original dataset)  1 – At least once a week (2 in original dataset) |
| Does not have access to television, radio, newspaper or internet. | Categorical-dichotomous | nomedia | Whether or not the respondent has access to any of the four forms of media considered in the study. Combined from v157, v159, v158, and s111. | 0 – No (if one to any of the following: v157, v158, v159, s111)  1 – Yes (if 0 to all of the following: v157, v158, v159, s111) |
| Number of lifetime sexual partners | Continuous | v836 | Number of sexual partners of the respondent as of the date of the interview. | As is |
| Number of lifetime sexual partners | Continuous | nosexp | Generated from v836 of the dataset. | As is. |
| Age of first sexual intercourse | Continuous | v531 | Imputed age of the respondent by the time she had her first sexual intercourse. | As is |
| Age of first sexual intercourse | Categorical | agefirst | Recoded from v531 of the dataset. | 11 - <15 y/o  17 – 15-19 y/o  22 – 20-24 y/o  27 – 25-29 y/o  39 – 30+ y/o |
| Knowledge of condom source | Categorical-dichotomous | v762az | Whether or not the respondent knows any legitimate source of condom. | 0 – Knows any source of condom  1 – Does not know any source of condom |
| Knowledge of condom source | Categorical - dichotomous | consource | Generated from v762az of the dataset. | 0 – Knows any source of condom  1 – Does not know any source of condom |
| Outcome | | | | |
| Ever tested for HIV | Categorical-dichotomous | v781 | Whether or not the respondent has ever been tested for HIV. | 0 – Never tested  1 – Yes; ever tested |
| Ever tested for HIV | Categorical - dichotomous | outcome | Generated from v781 of the dataset. | 0 – Never tested  1 – Yes; ever tested |
| Weights | | | | |
| Sampling weight | Continuous | v005 | Women’s individual sampling weight | As is |
| Sampling weight | Continuous | weight | Women’s individual sampling weight divided by 1000000 | As is |
| Stratifying variables and primary sampling units | | | | |
| Region | Categorical – 17 categories | v024 | Regions of the Philippines. | 1 – National Capital Region  2 – Cordillera Administrative Region  3 – Ilocos Region  4 – Cagayan Valley  5 – Central Luzon  6 – CALABARZON  7 – MIMAROPA  8 – Bicol  9 – Western Visayas  10 – Central Visayas  11 – Eastern Visayas  12 – Zamboanga Peninsula  13 – Northern Mindanao  14 – Davao  15 – SOCCSKSARGEN  16 – Caraga  17 - ARMM |
| Urban/Rural | Categorical – dichotomous | v025 | Urban/Rural | 1 – Urban  2 - Rural |
| Strata | Categorical – 33 categories | strata | Combined variables from v024 and v025. | 1 – National Capital Region (Urban)  2 – Cordillera Administrative Region (Urban)  3 – Cordillera Administrative Region (Rural)  4 – Ilocos Region (Urban)  5 – Ilocos Region (Rural)  6 – Cagayan Valley (Urban)  7 – Cagayan Valley (Rural)  8 – Central Luzon (Urban)  9 – Central Luzon (Rural)  10 – CALABARZON (Urban)  11 – CALABARZON (Rural)  12 – MIMAROPA (Urban)  13 – MIMAROPA (Rural)  14 – Bicol (Urban)  15 – Bicol (Rural  16 – Western Visayas (Urban)  17 – Western Visayas (Rural)  18 – Central Visayas (Urban)  19 – Central Visayas (Rural)  20 – Eastern Visayas (Urban)  21 – Eastern Visayas (Rural)  22 – Zamboanga Peninsula (Urban)  23 – Zamboanga Peninsula (Rural)  24 – Northern Mindanao (Urban)  25 – Northern Mindanao (Rural)  26 – Davao (Urban)  27 – Davao (Rural)  28 – SOCCSKSARGEN (Urban)  29 – SOCCSKSARGEN (Rural)  30 – Caraga (Urban)  31 – Caraga (Rural)  32 – ARMM (Urban)  33 – ARMM (Rural) |
| Primary sampling unit | Categorical | v021 | 800 primary sampling units/enumeration areas | 1 to 800 depending on sampling unit to which an observation belongs |
| Subpopulation Variable | | | | |
| Study population for multivariate analysis | Categorical - dichotomous | subpop | Indicates whether or not the respondent has missing data for multivariate analysis | 0 – Not included in the analysis  1 – Included in the analysis |
